# The Activity Patterns and Grouping Characteristics of the Remaining Goitered Gazelle (*Gazella subgutturosa*) in an Isolated Habitat of Western China

**DOI:** 10.3390/ani14162338

**Published:** 2024-08-14

**Authors:** Dezhi Peng, Zhirong Zhang, Junda Chen, Dehuai Meng, Yongliang Liang, Tianhua Hu, Liwei Teng, Zhensheng Liu

**Affiliations:** 1 College of Wildlife and Protected Area, Northeast Forestry University, Harbin 150040, China; pengdezhi1999@163.com (D.P.); zhang_zr@126.com (Z.Z.); chenjunda19980418@126.com (J.C.); mdehuai1995@163.com (D.M.); 2 Helan Mountains National Nature Reserve Management Bureau of Ningxia, Yinchuan 750014, China; lyongliang1@163.com (Y.L.); tianhuahu@126.com (T.H.); 3 Key Laboratory of Conservation Biology, National Forestry and Grassland Administration, Harbin 150040, China

**Keywords:** camera traps, ungulates, activity pattern, group, arid ecosystem

## Abstract

**Simple Summary:**

The daily activity rhythms of herbivores have significant ecological and conservation implications. Most studies have focused on predators and their prey, as well as sympatric carnivores or herbivores, with little attention given to ungulates in predator-free ecosystems. Using camera traps, we systematically studied the activity patterns and grouping characteristics of goitered gazelles in the Helan Mountains. Our findings reveal that goitered gazelles are crepuscular-like, with activity peaks occurring after dawn and before dusk. Their daily activity patterns and grouping characteristics vary across seasons, likely influenced by climatic conditions and resource availability. This research provides essential ecological insights for the restoration and conservation of goitered gazelle populations and underscores the importance of further behavioral studies on ecologically significant rare species in isolated habitats.

**Abstract:**

Wildlife activity patterns, which reveal the daily allocation of time and energy, are crucial for understanding survival pressures, adaptive strategies, and behavioral characteristics in different environments. Among ungulates, grouping behavior is a prevalent adaptive trait that reflects the population structure, mating systems, and life history strategies formed over long-term evolutionary processes. This study aimed to elucidate the daily activity patterns and grouping characteristics of the rare goitered gazelle (*Gazella subgutturosa*) in the Helan Mountains of western China from 2022 to 2023 using camera trap monitoring. With a total of 3869 camera days of effective trapping, we recorded 442 independent detections of goitered gazelles. The results revealed the following: (1) Goitered gazelle is primarily active during the day, showing an activity pattern similar to crepuscular animals, with two activity peaks occurring after dawn and before dusk. (2) Daily activity patterns showed both seasonal and sex differences. In the warm season, morning activity peaks occurred earlier, and afternoon peaks occurred later compared to the cold season. The overlap in daily activity patterns between females and males in the warm season was lower than that in the cold season, and this trend persisted throughout the year. (3) The number of times different types of groups were observed varied significantly, with single males and single females accounting for a larger proportion of all observed groups. There was no significant difference in group size across seasons, with groups typically consisting of 1–2 individuals. Our study provides detailed insights into the temporal ecology and population structure of goitered gazelles in arid and semi-arid ecosystems. This information will guide the identification of future conservation priorities and the development of management plans for the reserve.

## 1. Introduction

Daily activity patterns, developed through long-term evolutionary processes, are adaptive mechanisms that enable animals to synchronize with the temporal structure of their external environment [1]. These consistent behaviors ensure that physiological functions align with circadian rhythms, thereby optimizing survival and reproductive success [2,3]. In mammals, these daily activity patterns demonstrate significant plasticity in response to environmental variations, governed by physiological conditions such as endogenous clocks [4] and modulated by a range of abiotic and biotic factors [5,6]. Climate factors, including temperature, precipitation, and light levels, influence activity rhythms by affecting food availability and thermal regulation [7,8,9,10]. Interactions among species, such as competition, predation, and human disturbance, also shape the distribution of activity times throughout the day [11,12,13,14]. Understanding these activity patterns and their seasonal variations is vital for uncovering the intricate mechanisms driving habitat selection and ecological behavior [3]. With the increasing impact of tourism, grazing, and other human activities on wildlife habitats [15], research on these patterns is essential to elucidate the factors shaping wildlife behavior and the limits of their adaptability to environmental changes [16,17].

The population structure of ungulates, a fundamental aspect of demographic studies, is defined by group type, group size, and sex ratio. Maintaining a healthy population structure is crucial for the sustainable development of the population [18,19]. Ungulates exhibit grouping behavior in specific environments and times, which enhances their ability to detect and evade predators, access food resources, and find mates. This behavior, therefore, promotes survival and reproduction while reducing individual predation risk [20,21,22]. However, it can also lead to increased competition and a higher potential for disease transmission, ultimately having negative impacts on both individuals and the population as a whole [23].

Compared to traditional manual survey methods, infrared camera traps offer several advantages, including efficiency, continuity, low cost, and non-invasiveness [24]. With as few as 30–100 independent detections per species per season or year, these traps can be used to estimate activity patterns, making them reliable tools for assessing different wildlife behaviors [25]. Since there is no need to physically capture animals, camera traps provide a non-invasive alternative for wildlife surveys and research [26]. They help reduce human sampling bias and expand monitoring coverage [27]. Additionally, camera traps can achieve higher detection rates when monitoring rare and elusive species [28]. Over the past decade, numerous studies have utilized camera traps to investigate the daily activity patterns of mammals. Most of these studies have focused on predator–prey relationships involving large and medium-sized carnivores and their prey species [29,30,31,32], as well as intraguild interference competition and resource competition among animals [33,34,35,36,37]. However, there has been relatively little research on rare ungulates in arid, predator-free ecosystems.

The goitered gazelle (*Gazella subgutturosa*) is a typical ungulate of the Bovidae family which inhabits arid and semi-arid regions. Historically, its range extended widely from the Middle East to the Far East of Asia [38,39]. However, in recent decades, the population of goitered gazelles has declined dramatically [40,41,42,43] and their range has been shrinking due to habitat destruction, overgrazing, poaching, and climate change [44,45,46]. In 2016, the International Union for Conservation of Nature (IUCN) classified the species as vulnerable, with an estimated population of 42,000–49,000 individuals [47]. Moreover, the species has been declared extinct in several regions [48]. In China, the goitered gazelle is listed as a Class II protected wild animal, distributed in Xinjiang, Qinghai, Ningxia, Inner Mongolia, and Gansu [40]. In the Helan Mountains National Nature Reserve, there has long been a small and stable population of goitered gazelles [49]. As a typical “island” reserve, the Helan Mountains do not experience any immigration or emigration of wild ungulates, such as goitered gazelles. However, current research primarily addresses habitat suitability and genetic diversity of the Xinjiang populations [39,50,51,52,53,54], leaving a gap in knowledge regarding the behavioral ecology of the Helan Mountains population in China, especially in terms of daily activity patterns and grouping characteristics.

In the current study, we aim to fill this gap by quantifying the daily activity patterns and seasonal variations of goitered gazelles in the Helan Mountains through long-term infrared camera trap monitoring. We hypothesize that (i) like many wild ungulates [55,56,57,58], with 2–3 distinct daily peaks, goitered gazelles would exhibit diurnal activity; (ii) due to seasonal changes in climate, food resources, and differences in physiological cycles [59,60,61,62,63], there would be some seasonal and gender differences in their daily activity patterns; and (iii), both the group type and group size of goitered gazelles would exhibit seasonal variations, consistent with changes in their breeding cycle and foraging needs [64,65,66,67]. Assessing the temporal behavior patterns of wildlife is crucial for conservation and management, particularly for rare, elusive species or those inhabiting fragile ecosystems such as mountainous regions. This study will provide valuable data for managers, facilitating the effective conservation and scientific management of rare herbivores in arid and semi-arid ecosystems.

## 2. Materials and Methods

### 2.1. Study Area

**Figure 1:**
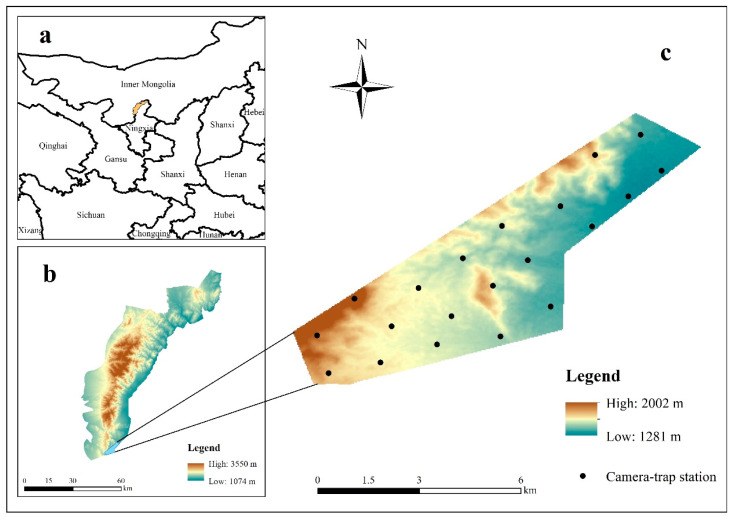
Location of the Helan Mountains National Nature Reserve in China (**a**); the Helan Mountains National Nature Reserve (**b**); distribution area of goitered gazelles and locations of camera traps (**c**).

The study area is located in the Helan Mountains National Nature Reserve in Ningxia of western China (38°19′–39°22′ N, 105°49′–106°41′ E), covering an area of 1935.36 km^2^ (Figure 1). Separated by the Yellow River, surrounding cities and deserts, this region is a transition zone between the temperate grasslands and deserts areas in China, with a typical temperate continental climate. The average annual temperature is −0.7 °C, with an average annual precipitation of 418.1 mm, mostly occurring from June to September [68]. The vegetation shows a distinct vertical distribution: a mountain grassland zone (1400–1600 m), mountain sparse forests and grasslands (1600–2000 m), mountain coniferous forests (1900–3000 m), and subalpine shrubs and meadows (3000–3556 m) [69]. In addition to goitered gazelles, this nature reserve is home to a variety of mammalian species, including snow leopards (*Panthera uncia*), alpine musk deer (*Moschus chrysogaster*), blue sheep (*Pseudois nayaur*), red deer (*Cervus elaphus*), red foxes (*Vulpes vulpes*), Eurasian badgers (*Meles meles*), and leopard cats (*Prionailurus bengalensis*) [70]. Due to prolonged human activities, the environment has gradually deteriorated, leading to weakened ecosystem functions and the severe destruction of natural habitats for wildlife. Currently, goitered gazelles are only distributed in a small area in the southeastern part of the reserve. The rutting season generally occurs from November to December each year, with a gestation period of 5–6 months [64,66].

### 2.2. Infrared Camera Trapping

From October 2022 to October 2023, we strategically deployed 20 infrared cameras across the study area. The deployment sites were selected based on evidence of goitered gazelle activity, such as proximity to water sources, trails marked by droppings, footprints, and resting spots, to comprehensively cover all distribution areas of goitered gazelles in the Helan Mountains. Detailed records of each camera’s location and the surrounding environment were meticulously maintained. To minimize redundant captures, the distance between any two camera traps was kept above 1 km. Cameras were relocated if they yielded minimal captures or if new signs of gazelle presence were detected elsewhere. The cameras were discreetly concealed and camouflaged to reduce human interference with site selection. Mounted on tree trunks or other stable structures at a height of 50–80 cm from the ground, the cameras were generally oriented northward to avoid direct sunlight. No baits were used to ensure the animals were captured in their natural state. The cameras operated continuously, capturing two photos and a 15 s video upon each trigger, with a 1 min interval between triggers, and sensitivity set to medium. Cameras were checked every three months to replace SD cards and batteries.

### 2.3. Analysis of Daily Activity Patterns

Upon data collection, each record’s information, including date, time, group size, gender, and other relevant details, was summarized and organized. All images of the same species captured at the same site within a 30 min interval were considered a single independent event [71]. Each independent detection event of the target species was regarded as a potential random sample from a continuous cyclic time distribution, representing the probability of capturing photos within any specific time interval throughout the day [72]. Considering the climatic characteristics of the Helan Mountains, the physiological cycle of the goitered gazelle, and seasonal variations in food resources, the year was divided into two seasons: the warm season (April to September) and the cold season (October to March of the following year). Consequently, data analysis was conducted at both annual and seasonal levels. Kernel density estimation was employed to analyze the daily activity patterns of goitered gazelles [72]. The temporal overlap between different seasons or sexes of goitered gazelles was calculated using an overlapping coefficient with the package “overlap” in R 4.3.2 [73]. The densityPlot function was used to draw plots of the kernel density of species, and the overlapEst function was used to estimate the overlap coefficient. When both sample sizes for pairwise comparisons were ≥75, Delta4 predicted values were chosen; when at least one sample size was <75, Delta1 predicted values were chosen. The overlap coefficient ranges from 0 to 1, with 0 indicating complete non-overlap and 1 indicating complete overlap. The temporal overlap was considered high if Δ > 0.75, intermediate if 0.50 < Δ < 0.75, and low if Δ < 0.50. The 95% confidence interval (95% CI) of the overlap coefficient was calculated by smoothed bootstrap resampling of 10,000 activity patterns [73,74,75]. The Hermans–Rasson test was used to assess whether the goitered gazelles exhibited a random activity pattern throughout the 24 h period [74]. It was computed through the package “circular”. The compareCkern function in the package “activity” was used to compare daily activity times across different seasons or genders [76].

### 2.4. Analysis of Grouping Characteristics

We carefully examined all images (photos and videos) captured by camera traps to identify goitered gazelles. Juveniles and subadults are easily recognized because of their small body size (and the small horn size for males), while adult males and adult females can be distinguished by the presence or absence of horns. Individuals who could not be clearly identified were not included in the statistical analysis. The groups of goitered gazelles captured in the images were categorized into six types: (1) single male; (2) single female; (3) male group, consisting of at least two adult males but no adult females; (4) female group, consisting of at least two adult females but no adult males; (5) female–offspring group, consisting of one adult female and at least one juvenile or one subadult but no adult males; and (6) male–female group, consisting of at least one adult male and one adult female. To analyze the differences in the number of observations among the various group types and their seasonal variations, chi-square tests were conducted. Additionally, Mann–Whitney tests were conducted to examine seasonal differences in group size among different group types [77]. We set the significance level at 0.05 for all tests. All data processing and analysis were performed using Excel 2021, Origin 2022, and R (version 4.3.2).

## 3. Results

**Figure 2:**
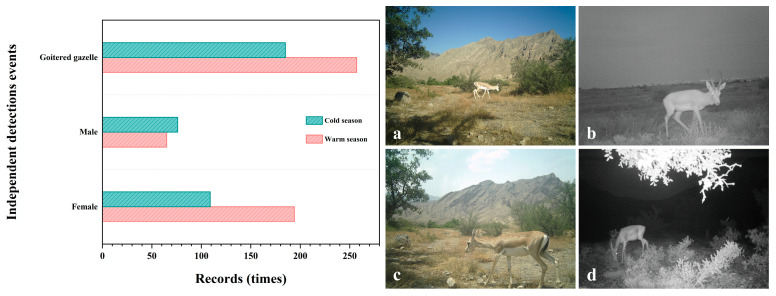
The number of independent detection events for goitered gazelles per season (**left**) and images of goitered gazelles captured by infrared camera traps during the day (**a**,**c**) and night (**b**,**d**) in the Helan Mountains, Ningxia, China from October 2022 to October 2023 (**right**).

Over the course of 3869 camera trap days, 442 independent detections of goitered gazelles were obtained. These detections were distributed across 12 different camera trap sites, with 185 detections recorded during the cold season and 257 during the warm season. Specifically, a total of 141 male detections were observed, with 76 occurring in the cold season and 65 in the warm season. In contrast, female detections totaled 303, with 109 recorded in the cold season and 194 in the warm season (Figure 2).

### 3.1. Daily Activity Pattern and Seasonal Differences of Goitered Gazelle

**Figure 3:**
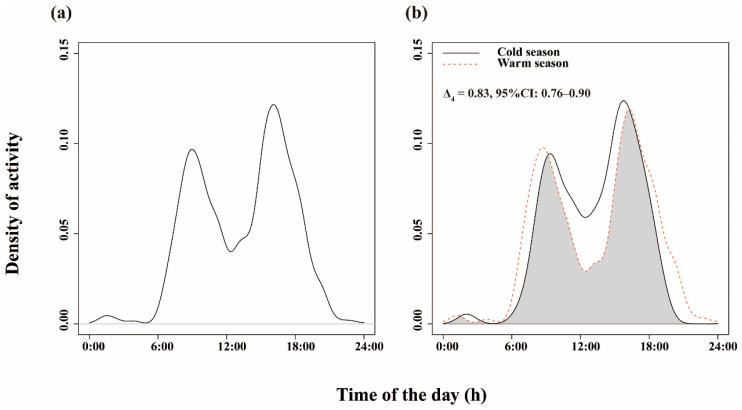
Daily activity pattern of goitered gazelles throughout the year (**a**) and in different seasons (**b**); 95% CI = 95% confidence intervals. In the cold season, sunrise occurs between 7:30 and 8:30, and sunset occurs between 17:30 and 18:30; while in the warm season, sunrise occurs between 5:00 and 6:00, and sunset occurs between 19:00 and 20:00.

The Hermans–Rasson test revealed a significant difference from random activity patterns in goitered gazelles (T = 189.24, *p* < 0.05). Throughout the year, goitered gazelles exhibited two pronounced activity peaks during daylight hours, specifically from 6:00 to 12:00 and 14:00 to 18:00 (Figure 3a). The overlap coefficient of daily activity patterns between the cold and warm seasons was high (Δ_4_ = 0.83; CI: 0.76–0.90) and the directions of the curves were roughly the same (Figure 3b). However, there was a significant difference in the activity times of goitered gazelles across different seasons (*p* < 0.05). Compared to the cold season, goitered gazelles increased their activity during the early morning (6:00–9:00) and night (18:00–24:00) in the warm season, while reducing activity at midday (10:00–14:00). Additionally, in the warm season, the morning activity peak occurred earlier, while the afternoon peak was delayed.

### 3.2. Gender Differences in Daily Activity Patterns across Different Seasons

**Figure 4:**
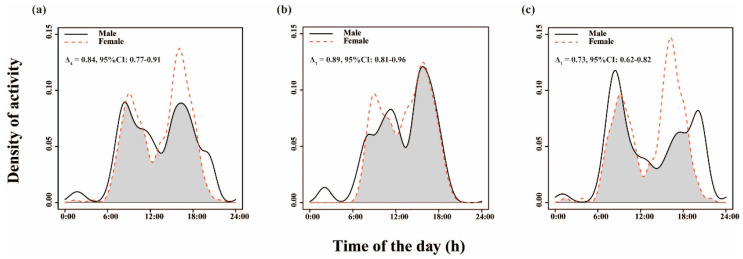
Gender differences in daily activity patterns of goitered gazelles throughout the year (**a**) and across seasons (cold season: (**b**); warm season: (**c**)); 95% CI = 95% confidence intervals.

Kernel density estimation analysis indicated a high overlap coefficient of daily activity patterns between males and females throughout the year (Δ_4_ = 0.84; CI: 0.77–0.91) (Figure 4a). However, there was a significant gender difference in the activity time (*p* < 0.05). Males exhibited higher activity density than females during the early morning (5:00–9:00) and post-dusk hours (19:00–4:00), although their peak activity periods were not entirely distinct. During the cold season, both sexes showed similar trends with a high overlap coefficient (Δ_1_ = 0.89; CI: 0.81–0.96) (Figure 4b). In contrast, the overlap coefficient was lower in the warm season (Δ_1_ = 0.73; CI: 0.62–0.82) (Figure 4c), and there was a significant difference in the activity time between males and females (*p* < 0.05).

### 3.3. Seasonal Differences in Group Type and Group Size

A total of 428 groups were identified and classified. The most common group type was single females (167 times, 39.02%), followed by single males (118 times, 27.57%), then female–offspring groups (81 times, 18.93%), female groups (39 times, 9.11%), male–female groups (16 times, 3.74%), with male groups occurring the least frequently (7 times, 1.64%). Chi-square tests revealed significant differences in the number of observations among different group types (χ² = 275.74, df = 5, *p* < 0.05).

**Figure 5:**
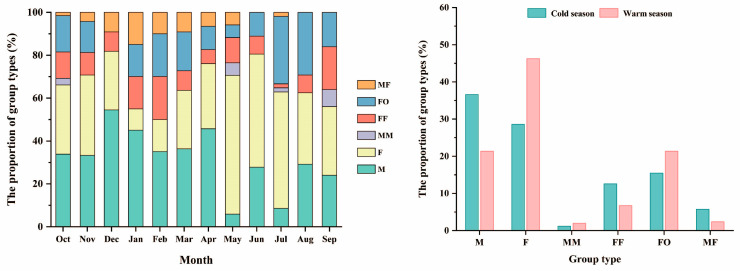
Proportion of different group types in different months (**left**) and seasons (**right**). M: single male; F: single female; MM: male group; FF: female group; FO: female–offspring group; and MF: male–female group.

Single males, single females, and female groups were observed every month and the proportion of individuals acting alone was more than 50%. Female–offspring groups were not observed only in December (Figure 5, left). There were pronounced seasonal differences in the number of observations for various group types (χ² = 26.31, df = 5, *p* < 0.05). The proportions of single males, female groups, and male–female groups were larger in the cold season, while the proportions of single females, male groups, and female–offspring groups were larger in the warm season. Male groups and male–female groups consistently accounted for lower proportions than other types in both seasons (Figure 5, right). 

**Figure 6:**
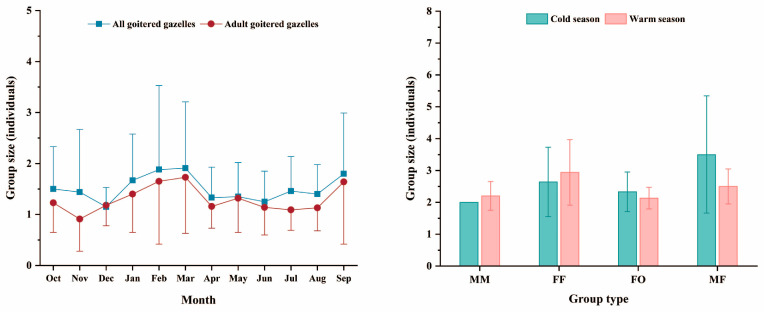
Group size of goitered gazelles in different months (**left**) and group size of different group types in different seasons (**right**). MM: male group; FF: female group; FO: female–offspring group; and MF: male–female group.

In this study, the group size of goitered gazelles recorded by the camera was 1.48 ± 0.86 (on average ± SD) individuals. During the cold season, the group size was 1.55 ± 1.02 individuals, with the largest group consisting of eight individuals. In the warm season, the group size was 1.43 ± 0.72 individuals, with the largest group consisting of five individuals. Regardless of the season, the majority of these groups consisted of 1–2 individuals (cold season: 89.51%; warm season: 91.53%), followed by groups of 3–4 (cold season: 9.09%; warm season: 7.63%), with groups of five or more being the least common (cold season: 1.40%; warm season: 0.83%). Mann–Whitney tests revealed no significant difference in group size between the cold and warm seasons (W = 24393, *p* > 0.05). The trends in group size for all gazelles and adult gazelles were roughly the same, with both peaking in March. However, the group size for all gazelles iwas the smallest in December, while the group size for adults was the smallest in November (Figure 6, left).

We also calculated the size of different group types in different seasons (Table 1). Male–female groups were the largest in both seasons. The size of female groups was larger in the warm season, while the size of female–offspring groups was larger in the cold season. In the warm season, the size of male groups was 2.20 ± 0.45 individuals, whereas in the cold season, it was recorded only twice, with two individuals in each group (Figure 6, right). Furthermore, there were no significant seasonal differences in the size of male, female, female–juvenile, and male–female groups (male group: W = 4, *p* > 0.05; female group: W = 146, *p* > 0.05; female–offspring group: W = 830.5, *p* > 0.05; male–female group: W = 40.5, *p* > 0.05).

## 4. Discussion

In this study, we analyzed the daily activity patterns of goitered gazelles in the Helan Mountains National Nature Reserve, China. The results revealed a bimodal activity pattern throughout the year, with peaks occurring around 9:00 and 16:00 in both cold and warm seasons, indicating that this species exhibits activity habits similar to crepuscular animals. Many ungulates are primarily active during the day, which helps them forage and quickly detect and avoid disturbances. Meanwhile, goitered gazelles’ crepuscular-like activity pattern allows them to avoid the midday heat and reduce water loss, especially during the warm season [78].

The circadian rhythm of animals is highly adapted to environmental conditions, influenced primarily by seasonal variations in climate [79], food availability [80], as well as physiological and life history cycles [81]. Wild animals exhibit distinct behavioral changes corresponding to variations in food quantity and quality across different seasons, with the photoperiod and temperature serving as key climatic factors influencing their activity patterns [78]. During the warm season, longer daylight hours, higher temperatures, and more abundant food resources due to plant growth are observed. However, the high temperature at noon limits the feeding of goitered gazelles, prompting them to rest and reduce activity to facilitate rumination and restore energy. Consequently, the morning activity peak occurs earlier, while the afternoon peak is delayed. The increased density of nocturnal activity in the warm season, especially among males, may also be an adaptation to higher daytime temperatures. In contrast, the cold season’s shorter daylight hours, cold climate, and thick snow cover create unfavorable conditions for foraging and activity. With low vegetation coverage, food resources become scarce. Goitered gazelle must extend their foraging time to obtain sufficient energy for thermoregulation, leading to reduced resting time at noon and a shift of the activity peak towards midday. The rutting season of goitered gazelles occurs from November to December each year, in which time male gazelles are vigilant and actively drive away other males to defend their territory and “harem” [82,83], resulting in a higher overlap coefficient of activity patterns between females and males in the cold season.

In the cold season, the early peak of activity for goitered gazelles shifted to around 9:30, with a lower peak value, while the late peak was higher. This suggests that in the cold season, goitered gazelles may reduce their early morning activity due to the delayed sunrise and lower temperature, preferring to be more active from the afternoon to dusk when temperatures are higher. Conversely, in the warm season, the late peak of activity occurred around 16:30, slightly higher than the early peak, indicating a preference for activity during the cooler afternoon hours. In addition, the differences in daily activity patterns between females and males may be related to the life history characteristics of different genders and their roles in raising offspring [84]. For example, adult females may tend to be more active after sunrise and before sunset as they need to care for their offspring and avoid various disturbances as much as possible [85,86].

Factors influencing the type and size of ungulate groups include population density, reproductive cycles, food resources, habitat openness, intraspecific competition, and predation risk. Previous studies have shown that the goitered gazelle can be categorized as an intermediate between social and solitary animals and are significantly closer to solitary animals, whose group sizes are independent of population density [67]. In this infrared camera survey, the main group types of goitered gazelles were single males and single females. Large carnivores such as wolves and lynxes, which were once natural predators of goitered gazelles in the Helan Mountains, became extinct in the 1980s [87]. On the other hand, the population of snow leopards is extremely low, and their range does not overlap with that of goitered gazelles. In addition, goitered gazelles have few suitable habitats in the Helan Mountains, resulting in a small distribution range and no seasonal migration. The lack of predation risk and the limited range of their activities may both account for their predominantly solitary behavior or the formation of small groups. Male groups were observed the least frequently, possibly due to a stronger sense of competition among males. In the cold season, there were increases in the proportions of single males, female groups, and male–female groups, while the proportions of single females, male groups, and female–offspring groups decreased, which might be related to various reproductive behaviors during the rutting season. Males will establish breeding territories along the daily movement routes of females, mate with multiple females, and attempt to confine them within the territory while preventing other males from contacting them [82,83]. During the warm season, pregnant females choose to give birth in hidden and safe locations, after which females need to raise and protect their offspring [85,86], thus potentially leading to successive increases in the proportions of single females and female–offspring groups. The abundance of food resources and lack of competition for mates may make male groups more likely to appear in the warm season.

We also found that there are no significant seasonal differences in the group size of goitered gazelles or in the group size of different group types. The groups usually consist of 1–2 individuals, which may be related to the feeding ecology of the goitered gazelle, as it is a highly selective ungulate with a small body size [65]. These results suggest that forming small groups may be a key characteristic of goitered gazelles in the Helan Mountains. 

## 5. Conclusions

Utilizing infrared camera technology, this study investigated the daily activity pattern and grouping characteristics of rare goitered gazelles in the arid and semi-arid areas of the southern Helan Mountains, Ningxia, as well as their seasonal variations. Our findings reveal distinct tendencies in activity times and group composition between cold and warm seasons, influenced by climate conditions and food resources. However, we did not quantitatively analyze the driving mechanisms behind these changes, which warrants further research. We recommend improving the habitat by maintaining the grassland ecosystem, protecting water sources, and reducing human disturbance to specifically promote the recovery of the goitered gazelle population in this region.

## Figures and Tables

**Table 1:** Maximum, minimum, mean, and standard deviation of group size for different group types and of males and females in male–female groups across different seasons.

Group Type	Cold Season				Warm Season			
	Max	Min	Mean	SD	Max	Min	Mean	SD
male group	2	2	2.00	0.00	3	2	2.20	0.45
female group	6	2	2.64	1.09	5	2	2.94	1.03
female–offspring group	4	2	2.33	0.62	3	2	2.13	0.34
male–female group	8	2	3.50	1.84	3	2	2.50	0.55
males in male–female group	2	1	1.10	0.32	2	1	1.17	0.41
females in male–female group	4	1	2.00	1.05	1	1	1.00	0.00

## Data Availability

The datasets used in this study are available from the corresponding author upon reasonable request.
